# Study on multi field coupling numerical simulation of nitrogen injection in goaf and fire-fighting technology

**DOI:** 10.1038/s41598-022-22296-9

**Published:** 2022-10-17

**Authors:** Wei Wang, Yun Qi, Jiao Liu

**Affiliations:** 1 School of Coal Engineering, Shanxi Datong University, Datong, 037000 People’s Republic of China; 2 School of Emergency Management and Safety Engineering, China University of Mining & Technology, Beijing, 100083 People’s Republic of China; 3 China Safety Science Journal Editorial Department, China Occupational Safety and Health Association, Beijing, 100011 People’s Republic of China

**Keywords:** Chemical engineering, Chemical engineering

## Abstract

In order to effectively prevent the spontaneous combustion of residual coal in goaf, taking the 10,101 fully mechanized top coal caving face of Baozigou coal mine as research object, the multi-field coupling numerical model of nitrogen injection in goaf is established. The FLUENT software is used to study the variation law of spontaneous combustion zone in goaf under dynamic mining of working face with different nitrogen injection parameters, determining the range of spontaneous combustion zone in stable stage. The fitting curve between nitrogen injection parameters and width of spontaneous combustion zone in goaf is obtained. Results show that with the increase of nitrogen injection depth from 10 to 60 m, the width of spontaneous combustion zone in goaf begins to decrease gradually, yet the width of spontaneous combustion zone tends to expand after more than 40 m. When the nitrogen injection location is 40 m, the spontaneous combustion zone width decreases from 49 to 22 m as the nitrogen injection volume increases from 500 to 1000 m^3^/h. Nitrogen injection continuously reduces the area of high temperature zone and temperature extreme value. When the nitrogen injection parameter is set to (40 m–1000 m^3^/h), temperature extreme value decreases by 308.85 K compared with that without nitrogen injection. When the nitrogen injection parameter is (40 m–690 m^3^/h), it can meet the inert cooling requirements of goaf. The width of spontaneous combustion zone is 31 m and the temperature extreme value is 309.95 K at the moment. Finally, engineering application of the fire prevention technology combining shot-off loss wind and nitrogen injection is used to test effect of spontaneous combustion prevention and verify accuracy of nitrogen injection simulation. CO concentration at the measuring point 1, 3 and 5 are reduced to 0 × 10^−3^‰, 2 × 10^−3^‰ and 1.2 × 10^−3^‰, and temperature are reduced to 295.15 K, 296.15 K and 295.65 K respectively. It shows that the spontaneous combustion of residual coal in goaf has been successfully controlled.

## Introduction

Spontaneous combustion of residual coal in goaf is a kind of mine fire caused by some complex factors, accounting for about 85% of the total underground fires^1,2^. With the continuous improvement of coal mining technology, coal mine production takes on a trend of high yield, high efficiency and intelligence, and there are more and more high mining height coal mining faces. Although the production efficiency of mine has greatly increased, there are also some problems, such as serious coal residue in goaf, high caving height, large air demand in working face, higher air leakage and slower propulsion speed, resulting in further increase of the risk of coal spontaneous combustion in the goaf^3,4^. Spontaneous combustion often occurs in the deep part of the goaf at a certain distance from the working face. However, most spontaneous combustion just smokes without open fire, so it’s difficult to determine the location of fire source^5^. Spontaneous combustion in the goaf will release a large amount of toxic gas and cause disasters such as gas and coal dust explosion, which will endanger the life safety of personnel and even lead to the shutdown or closure of the working face^6,7^. Therefore, the prevention and control of spontaneous combustion of residual coal in goaf is an urgent and significant topic.

The spontaneous combustion of residual coal in goaf has traditionally been a major mine hazard concerning scholars in the field of coal mine safety, and key research has been carried out on spontaneous combustion prevention and control techniques in goaf^8–10^. Zhang Xiaoqiang et al.^11^ by injecting isolation material through the interval between the hydraulic support frames to form a row of discontinuous isolation blocking zones, a dynamic pressure injectionisolation technology for collaborative control of coupling thermodynamic disaster in goaf in special mining period was proposed and the isolation parameters of the field goaf were designed. Fan Cheng et al.^12^ took the 6306 working face of the Tangkou coal mine as an engineering example, used the discrete element method to quantitatively simulate the porosity distribution in goaf from a microscopic perspective. Magdalena Tutak^13^ determined the critical values of seepage velocity and oxygen concentration in the goaf, established a mathematical model of coal spontaneous combustion, and simulated the distribution of spontaneous combustion zone in the goaf, which can be used to guide to resolve problem of coal spontaneous combustion in the goaf at the working face of U-shaped ventilation system in longwall mining. Yu Xu et al.^14^ established a coupled mathematical model of gas and coal spontaneous combustion, by which a composite hazard of gas and coal spontaneous combustion in the goaf was analyzed and the location of gas explosion and spontaneous combustion zone in the goaf was predicted by using multivariate functions. Hui Zhuo et al.^15^ established a numerical model of discrete fractures in porous media in the goaf of shallow buried coal seam, and introduced the model into FLUENT software to numerically simulate the O_2_ concentration field, CO concentration field and air velocity field in the goaf of Bulianta coal mine, and the simulation results were in good agreement with the actual measured data. Wei Liu et al.^16^ established a multi-field coupled transient mathematical model to simulate the effect of external mining parameters on spontaneous combustion during the stopping period. It can be seen from the simulation that the time required for coal spontaneous combustion during the stopping period can be effectively increased by reasonable adjustment of mining parameters. Chu Tingxiang et al.^17^ proposed an improved dynamic simulation of spontaneous combustion in the goaf to simulate the change law of each physical field in the goaf during the mining process of the working face, which can realize the inversion of coal spontaneous combustion process from a dynamic perspective. Jian Zhang et al.^18^ established a three-dimensional transient CFD model to analyze the temperature field change law in the goaf and formulate a targeted inert gas injection fire-fighting plan. Dongjie Hu et al.^19^ took the 11,101 working face of Qipanjing mine as the research object to explore the dynamic change law of the spontaneous combustion of the residual coal in the gob-side entry retaining goaf area. The dynamic distribution of the flow field in the gob-side entry retaining goaf was simulated with different advancing positions and air leakage at the working face, and fire prevention measures via grouting in the return air lane side and nitrogen injection in the retaining lane side were put forward. Liming Yuan et al.^20^ used CFD simulations to optimize the nitrogen injection scheme in goaf by establishing a mathematical model for nitrogen injection in goaf, which improved the inerting efficiency. Ma Dong et al.^21^ used COMSOL software to simulate nitrogen injection simulations and indicated an approximate power exponential relationship between the spontaneous combustion zone width and the amount of nitrogen injection. Luo Xinrong et al.^22^ used FLUENT software to simulate and study the effect of different extraction methods and nitrogen injection on spontaneous combustion of residual coal in goaf, pointing out that nitrogen injection process parameters had a significant impact on fire protection effect, and that nitrogen injection amount and nitrogen injection spacing should be properly controlled and adjusted. Jia Baoshan et al.^23^ took 1303 fully mechanized top coal caving face of Jinniu coal mine as the research object, and used numerical calculation software to study the distribution law of spontaneous combustion zone in goaf with different nitrogen injection parameters. The results showed that the most suitable nitrogen injection location was 30 m in goaf. It was concluded by Origin software that nitrogen injection amount was exponential relationship with the width of spontaneous combustion zone, thus obtaining optimal nitrogen injection amount. Guoqing Shi et al.^24^ took Liangbaosi coal mine working face as the research object, established a mathematical model of temperature field in goaf and verified its correctness. Then the model was used to simulate the variation law of temperature distribution in goaf changing with time when liquid nitrogen was injected at different locations, which provided a quantitative analysis method for preventing the coal spontaneous combustion in goaf with liquid nitrogen injection. Qi Yun et al.^25^ studied the influence of different nitrogen injection locations on the distribution of spontaneous combustion "three zones" in goaf by means of calculation software simulation. The results showed that gradually deepening nitrogen injection location had a significant impact on the lower limit of spontaneous combustion zone, but not on the upper limit of spontaneous combustion zone. Through optimization, the optimal nitrogen injection location reduces the width of spontaneous combustion zone to 28 m. However, above numerical simulations of nitrogen injection in goaf are static simulations or transient simulations of the model with a constant calculation area, which are static numerical and two-dimensional models of nitrogen injection in goaf. These simulations only analyze the change of single physical field in goaf without considering the coupling effect of multiple physical fields. It is difficult to comply with actual process of dynamic mining in working face. To further accurately understand inerting effect of nitrogen injection in goaf , it is necessary to study the influence law of nitrogen injection parameters on residual coal spontaneous combustion by dynamic numerical simulation methods from multi-field coupling perspective.

In view of this, taking the 10,101 fully mechanized top coal caving face of Baozigou coal mine as the research background, the author determines the distribution area of coal spontaneous combustion "three zones" through the bundle tube monitoring in goaf. The FLUENT software is used to establish a three-dimensional dynamic nitrogen injection multi-field coupling numerical model in goaf to simulate and study the changes of air leakage flow field, oxygen concentration field and temperature field in goaf with different nitrogen injection parameters. It analyses influence law of nitrogen injection on distribution of spontaneous combustion "three zones" in dynamic scale and verifies the correctness of simulation with measured data in goaf. The combination of shot-off loss wind and nitrogen injection is proposed as a fire-fighting technique to achieve scientific treatment of coal spontaneous combustion in goaf of 10,101 fully mechanized top coal caving face.

## Bundle tube monitoring of “three zones” in goaf

### Overview of working face

The 10,101 fully mechanized top coal caving face of Baozigou coal mine, located in mining coal seam 9 + 10 + 11 of the first mining area, is the first working face of the mine. The ventilation mode of the working face is U-shaped ventilation. The actual air supply capacity of the working face reaches 1300 m^3^/min. The inclined length of the working face is 150 m, with a strike length of 1205 m for the inlet airway and 1235 m for the return airway. Mining method of the working face is fully mechanized top coal caving technology. Maximum mining height of the working face shearer is 2.5 m. Height of the top coal caving is 2.97 m. Mining and caving ratio is 1:1.2. Advancing speed is 3.6 m/d. The coal seam has a slope of 6–13°, Grade I spontaneous combustion coal seam, with shortest coal spontaneous combustion period of 27 d. Its coal dust explosion index is 46.84%, indicating the possibility of explosion.

### Distribution of spontaneous combustion "three zones" in goaf

The "three zones" of residual coal spontaneous combustion in goaf is objective. However, due to lack of technical means to measure “three zones” and complex characteristics of fully mechanized mining face in goaf, it is difficult to achieve precise delineation when monitoring the site^26^. Spontaneous combustion of residual coal in goaf is the result of the combination of continuous oxygen supply due to air leakage from working face and heat storage from coal oxidation^27,28^. Main methods for classifying the "three zones" of spontaneous combustion in goaf are oxygen concentration method, air leakage velocity method and temperature rise rate method, which corresponds to classification indexes for classifying the spontaneous combustion zones of oxygen concentration in the range of 7% to 18%, air leakage velocity in the range of 0.1 m/min to 0.24 m/min and the temperature rise rate ΔT ≥ 1 ℃/d in goaf^29^.

**Figure 1:**
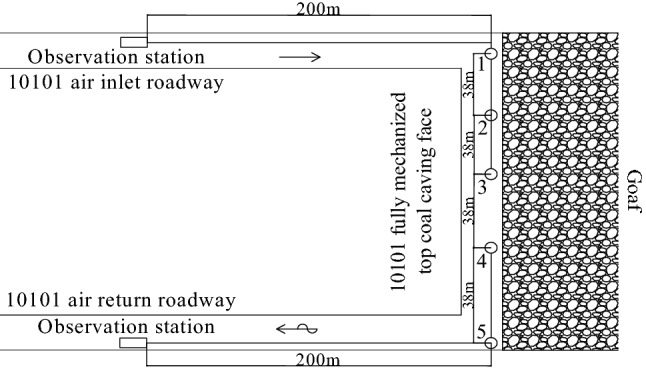
Layout of beam tube and temperature sensor.

**Figure 2:**
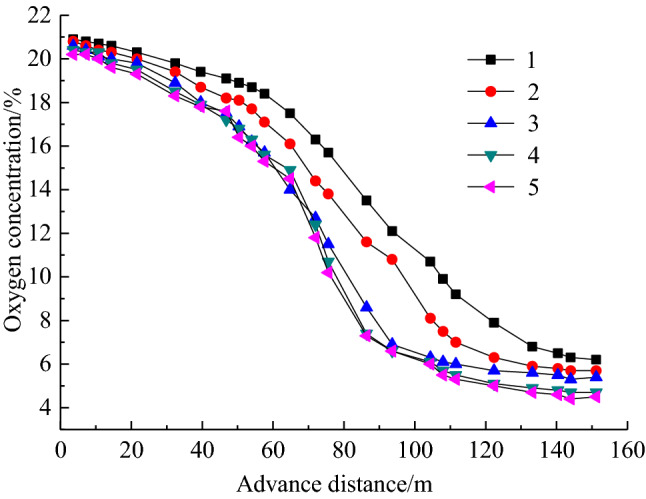
Change law of oxygen concentration with working face advancing.

The bundle tube and temperature measurement system were installed at the 10,101 fully mechanized top coal caving face, inlet and return air roadway, respectively, to measure and count the gas content and temperature data in the goaf during forward mining. The bundle tube and temperature measurement system were installed along the working face behind the hydraulic support 5 measuring points were arranged from air inlet roadway to air return roadway in turn. Observation stations were arranged at the 200 m outside the measurement points, with measurement point numbers 1 ~ 5 and a spacing of 38 m. The specific layout of measuring point is shown in Fig. 1. Through continuous testing, variation curve of oxygen concentration at every measuring point with the advancement of the working face is shown in Fig. 2. It is known from Fig. 2 that the oxygen concentration on side of air inlet roadway is higher than that on side of air return roadway, which is affected by the air leakage from the U-shaped ventilation system of the working face to the goaf. According to the division standard of oxygen concentration in the range of 7% ~ 18%, division results of coal spontaneous combustion "three zones" in goaf are shown in Table 1. From Table 1, the width of spontaneous combustion zone is 70 m on the inlet side, 55 m in the middle and 52 m on the return side, which shows a reduction in the width of the zone along the inclination of the working face.

**Table 1:** Range of spontaneous combustion "three zones" in goaf of different measuring point.

measuring point	cooled zone/m	spontaneous combustion zone/m	suffocating zone/m
1	0 ~ 62	62 ~ 132	> 132
2	0 ~ 52	52 ~ 112	> 112
3	0 ~ 40	40 ~ 95	> 95
4	0 ~ 38	38 ~ 93	> 93
5	0 ~ 38	38 ~ 90	> 90

**Figure 3:**
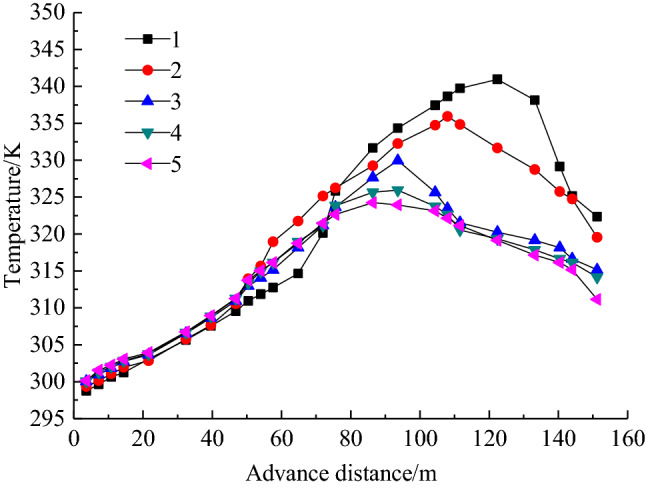
Law of temperature change of each measuring point.

The change curve of temperature in goaf with advancement of the working face is shown in Fig. 3. According to Fig. 3, the temperature in goaf is similar to the distribution characteristics of oxygen concentration, which shows that temperature of the inlet air side is higher than the return air side. Obviously, the change of temperature is greatly affected by the oxygen concentration, because the oxidation and heating of residual coal requires a suitable oxygen supply environment. Temperature rise rate method is used to divide the “three zones” by statistical temperature data. Its distribution range is slightly lagging behind that of the oxygen concentration, but is basically identical with the results, which fully verify the above analysis.

## Model establishment and solution conditions

### Three-dimensional dynamic goaf geometric model

**Figure 4:**
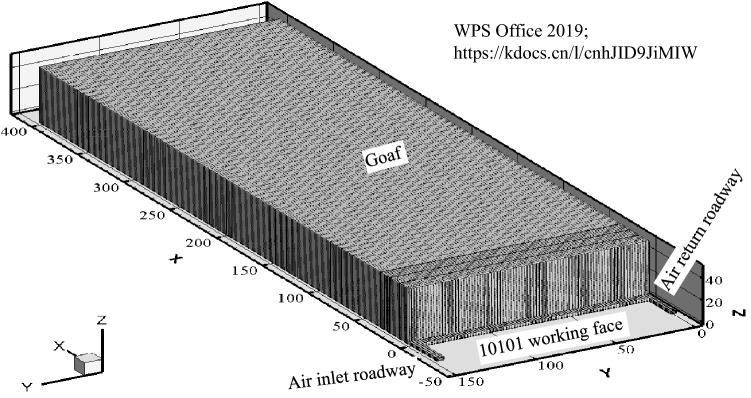
3D dynamic geometric model of goaf.

The size of the geometric model is determined by the actual situation of 10,101 fully mechanized top coal caving face of Baozigou coal mine. The length of working face is 150 m, and the height is the total height of caving zone and fracture zone of 50 m. The length of goaf is taken to be 400 m as an illustration. In order to reflect the actual situation of the ventilation resistance of the working face and simplify the model, the section of working face is 6 m × 3.2 m and the section of air return roadway is 3.6 m × 3.2 m and the section of air inlet roadway is 4.5 m × 3.2 m, with the length of 25 m. The three-dimensional geometric model is shown in Fig. 4.

### Boundary conditions and calculation parameters setting

The solution is based on a unit time step of 1 d, with an average daily advance speed of 3.6 m/d as the increasing rate of goaf length. With the advancement of working face, the goaf is gradually extended. The grid is automatically updated by the FLUENT software's dynamic grid model, thus reflecting the continuous advance of working face. Space moving coordinate system forms a new grid at a 3.6 m/d advance rate. According to the characteristics of dynamic grid model, the boundary conditions move as the working face advances dynamically. The temperature of the newly emerged coal rock is set as the initial temperature, after which the temperature continues to rise with the compound action of coal-oxygen. The functions of porosity of the goaf and the advancing speed of the working face is established and imported into FLUENT software to start the non-stationary calculation, thus presenting the variation of the goaf in space and time.

The dynamic advancement of the working face causes continuous spatial expansion of the goaf. The porosity of the extraction zone is variable in both time and space, although there is a general pattern. Supported by the coal wall of the inlet and return tunnel, the porosity of the goaf is greater than the porosity of the central location. In the meantime, due to the continuous compaction of the coal rock, the porosity is smaller as it goes deeper into the goaf. The porosity distribution function of the goaf is the following^30^:

1 $$n = n_{x} n_{y} n_{z} = \left\{ \begin{gathered} \left( {0.2e^{ - 0.0223x} + 0.1} \right) \cdot \left( {e^{ - 0.15y} + 1} \right) \cdot 1.05^{z} \left( {y \le {\text{L}}/2} \right) \hfill \\ \left( {0.2e^{ - 0.0223x} + 0.1} \right) \cdot \left( {e^{{ - 0.15\left( {L - y} \right)}} + 1} \right) \cdot 1.05^{z} \left( {y{\text{ > L}}/2} \right) \hfill \\ \end{gathered} \right.$$

where *n*_x_, *n*_y_, *n*_z_ refer to porosity along Y-axis, X-axis and Z-axis respectively, %; L is the length of the working surface, m.

When selecting the main calculation conditions and parameters, it is necessary to follow the actual situation on site. The air inlet roadway of the working face is set as the inlet boundary. The air return roadway is set as outflow. The nitrogen injection port is set as the velocity inlet and the concentration of nitrogen is taken to be 97%. The temperature of the working face boundary can be obtained by actual measurement and belongs to the first type of boundary conditions. The amount of heat exchange between the gas in the goaf and the coal wall is very small. Assuming that the heat flux on the boundary is 0, it can be treated as an adiabatic boundary^31^. The initial temperature of the goaf is 300.05 K. The actual measured airflow temperature in the air inlet roadway is 291.75 K. The oxygen concentration is 20.9% and the actual wind speed at the working face is 1.62 m/s. The average air density is 1.225 kg/m^3^. The air viscosity coefficient is taken to be 1.7894 × 10^−5^ kg/(ms). The diffusion coefficient of gas is 2.88 × 10^−5^ m^2^/s and the loosening coefficient is set to 1.5, The thickness of the residual coal in the goaf is 0.86 m.

The basic assumptions are proposed as the following:

1. Since the goaf caving band is random, the porous media in the goaf is isotropic and continuous.
2. The influence of periodic weighting on the goaf is ignored, that the seepage characteristics of air in the goaf are only related to its depth, and that the overlying rock body is not involved in the reaction, only existing seepage and heat transfer.
3. As the working face area is in an open tunnel, the air flow is in a steady turbulent state. The flow of mixed gases in the goaf is considered to be unsteady, incompressible, and with heat transfer.
4. The gas leaking into the goaf is considered to be the same temperature as the working face, and the gas temperature at the same coordinate position node in the goaf is equal to the solid temperature, which satisfies the thermal equilibrium between the risen coal rock and the gas.
5. The effect of convection and heat conduction on heat transfer is just considered, ignoring thermal radiation and other phenomena and the effect of water phrase change on the heat and mass transfer process of coal spontaneous combustion in the process of coal spontaneous combustion.

When using the numerical simulation results to divide the spontaneous combustion "three zones" in the goaf of a fully mechanized top coal caving face, it is necessary to consider the distribution of the oxygen concentration field and the air leakage velocity field in the goaf at the same time. If the oxygen concentration *C* can be combined with the distribution of the air leakage velocity *v*, it will be more in line with the actual distribution of the coal leftover spontaneous combustion area in the goaf of the fully mechanized caving face^32,33^. In order to reasonably divide the "three zones" of spontaneous combustion in the goaf of 10,101 fully mechanized top coal caving face, the combination method of air leakage velocity and oxygen concentration (*v* ≤ *v*_max_ ∩ *C* ≥ *C*_min_) is used. Among the method, *v*_max_ is the upper air leakage velocity that satisfies the oxidative heat storage condition of the residual coal in the goaf, and *C*_min_ is the lower oxygen concentration that causes oxidative spontaneous combustion of the residual coal^34^.

## Effect of nitrogen injection parameters on distribution of "three zones" in goaf

### Effect of nitrogen injection location on the "three zones"

**Figure 5:**
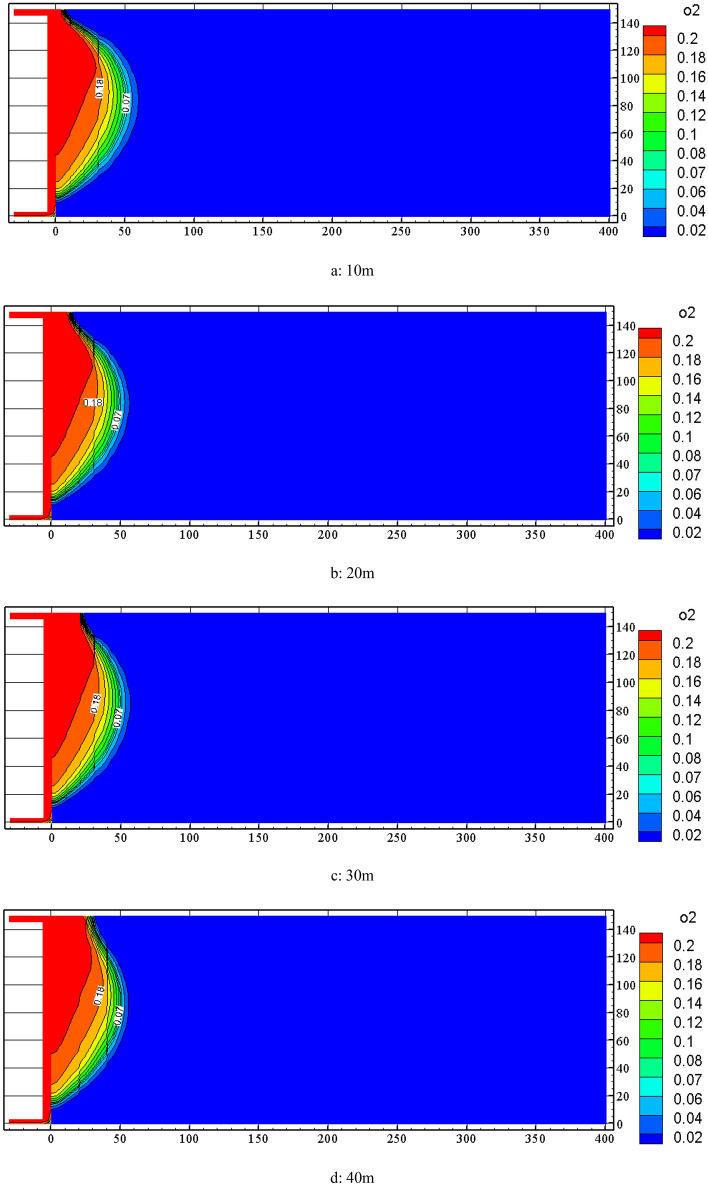

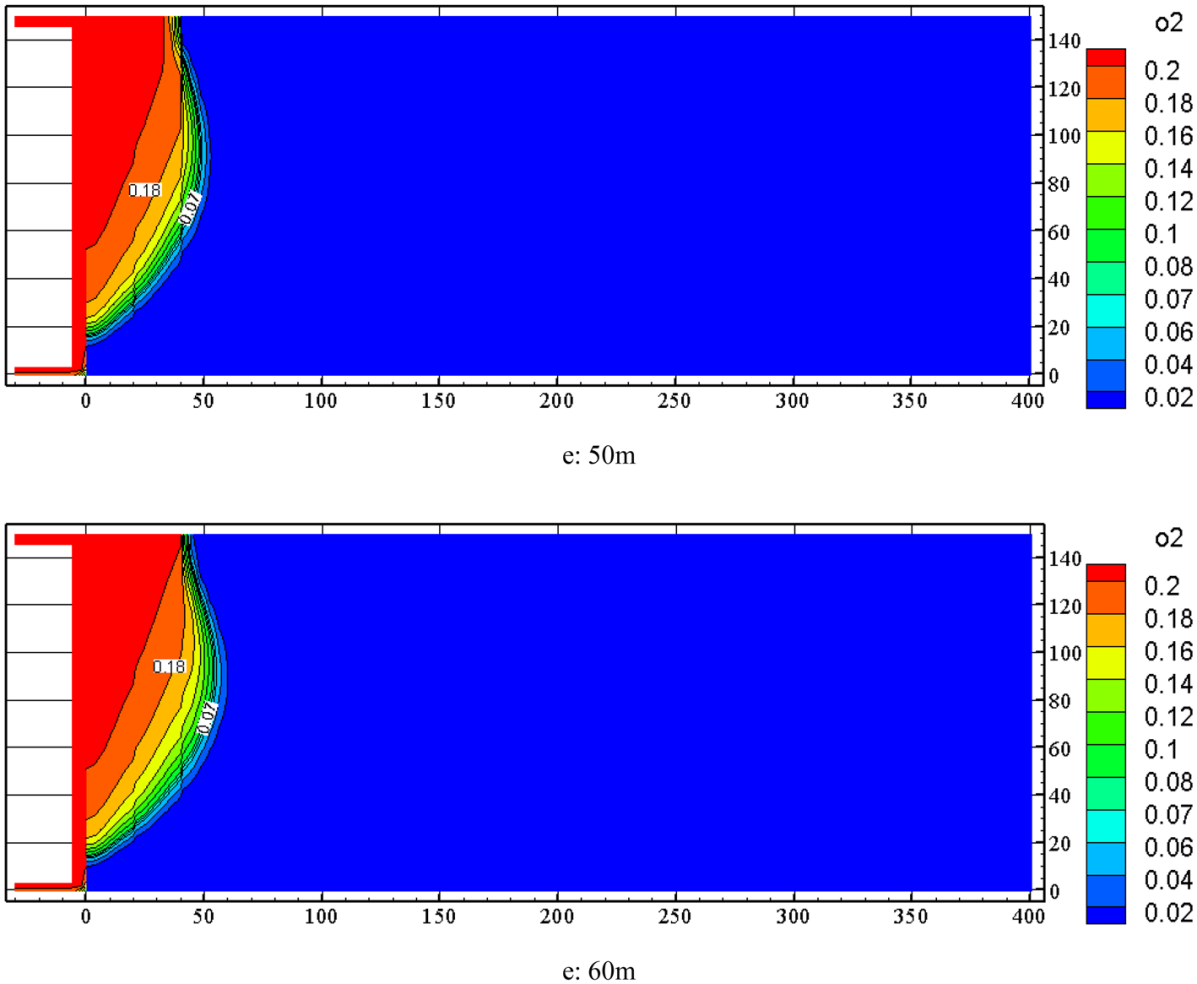
Distribution of oxygen concentration in goaf under different injection nitrogen location.

With the change of nitrogen injection location, the distribution of spontaneous combustion "three zones" in the goaf will also change. In order to analyze the effect of nitrogen injection location, FLUENT software was used to simulate the distribution changes of oxygen concentration field distribution in the goaf at the locations of 10 m, 20 m, 30 m, 40 m, 50 m and 60 m when the nitrogen injection volume was 700 m^3^/h (calculated from daily coal production at the working face), and the results are shown in Fig. 5. From Fig. 5, the location change of nitrogen injection will change the oxygen concentration distribution in the goaf. Since nitrogen is injected into the goaf from the air inlet side, the oxygen in the inlet air is displaced by nitrogen and has a good inerting effect, resulting in a lower oxygen concentration on the air inlet side than that in the middle and return air side and the rate of decline is fast. With the increasing depth of nitrogen injection, the 18% and 7% oxygen concentration contours in the goaf will also change. At the same time, the distance between the two keeps shrinking, but when the nitrogen injection depth exceeds 40 m, the distance increases again.

**Figure 6:**
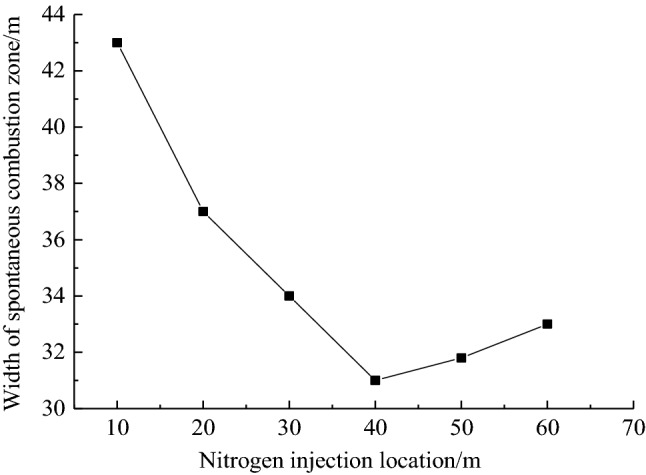
Goaf spontaneous combustion zone width with injection nitrogen location change.

Combined with the simulation results of the air leakage velocity distribution in the goaf at nitrogen injection locations of 10 m, 20 m, 30 m, 40 m, 50 m and 60 m, the Origin software was used to obtain the width of the spontaneous combustion zone and the location of nitrogen injection curve on the basis of the standard division (*v* ≤ *v*_max_ = 0.24 m/min ∩ *C* ≥ *C*_min_ = 7%), as is shown in Fig. 6. From Fig. 6, the corresponding spontaneous combustion zone widths are 43 m, 37 m, 34 m, 31 m, 31.8 m and 33 m respectively when nitrogen injection locations are 10 m, 20 m, 30 m, 40 m, 50 m and 60 m. The spontaneous combustion zone widths are reduced to 59%, 51%, 47%, 42%, 43.6% and 45% of those in the uninjected nitrogen condition. As the nitrogen injection port moves toward the depth of the goaf, the width of the spontaneous combustion zone in the goaf begins to shrink gradually. The width of the spontaneous combustion zone does not continue to decrease but increases when the nitrogen injection port penetrates deeper than 40 m into the goaf. This is because the nitrogen injection port go excessively deep into the goaf, and the inerting effect of nitrogen injection on the shallow part of the goaf is not obvious. Through the above analysis, the depth of nitrogen injection in the goaf should be 40 m ~ 50 m, taking 40 m as the most suitable nitrogen injection location.

### Effect of nitrogen injection volume on the “three zones”

**Figure 7:**
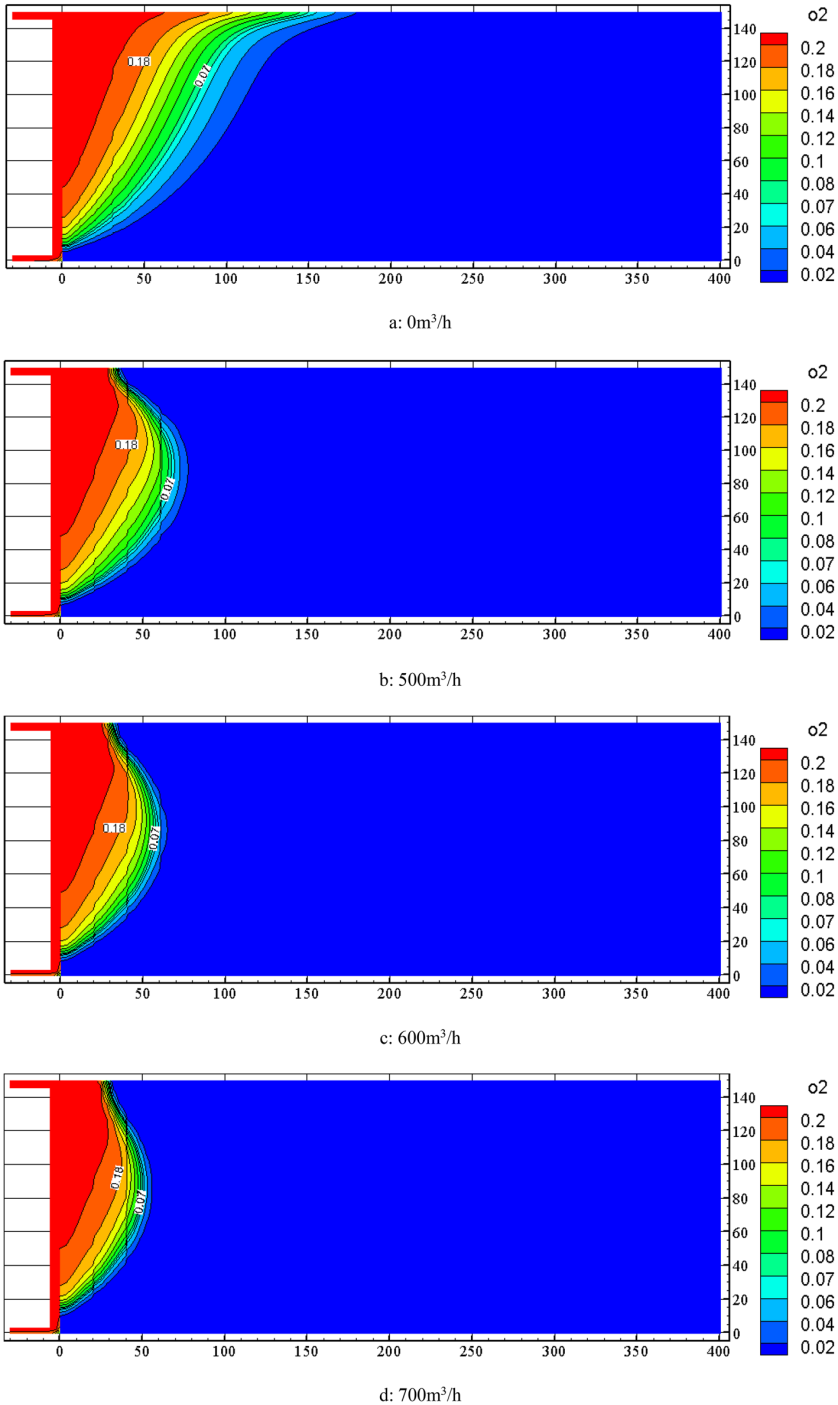

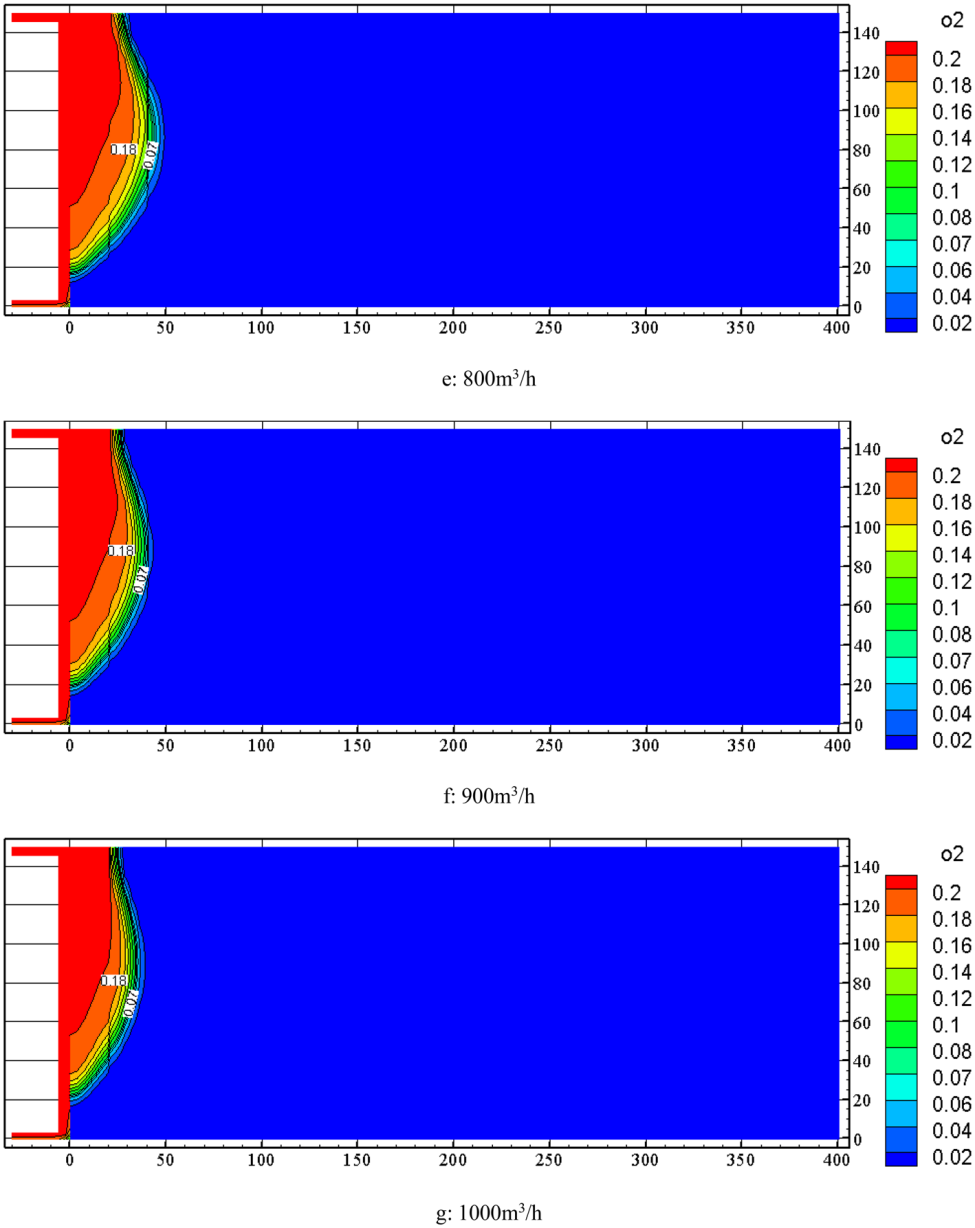
Distribution of oxygen concentration in goaf under different injection nitrogen volume.

When the nitrogen injection depth is 40 m, the FLUENT software is used to simulate the distribution change of the oxygen concentration field in the goaf under the conditions of 0 m^3^/h, 500 m^3^/h, 600 m3/h, 700 m^3^/h, 800 m^3^/h, 900 m3/h, and 1000 m3/h. The results are shown in Fig. 7. From Fig. 7 to know, the change in nitrogen injection volume causes the distribution change of oxygen concentration in the goaf. With the continuous increase of nitrogen injection volume, the distribution range of oxygen concentration in the goaf gradually narrows, the 18% and 7% oxygen concentration contour moves to the working face, and the distance between the two is continuously reduced.

By combining with the simulation results of air leakage velocity distribution in the goaf under the cases of 0 m^3^/h, 500 m3/h, 600 m3/h, 700 m^3^/h, 800 m^3^/h, 900 m^3^/h and 1000 m^3^/h of nitrogen injection volume, the width of spontaneous combustion zone based on the standard division (*v* ≤ *v*_max_ = 0.24 m/min ∩ *C* ≥ *C*_min_ = 7%) is obtained as shown in Table 2. As can be seen from Table 2, the 0.24 m/min air leakage velocity contour is not very significantly affected by the nitrogen injection volume, and only moves 9 m along the recovery direction. While the 7% oxygen concentration contour is relatively more significantly affected by the nitrogen injection volume, moving about 36 m along the recovery direction. As the nitrogen injection volume increases from 500 m^3^/h to 1000 m^3^/h, the width of the spontaneous combustion zone in the goaf is reduced from 49 to 22 m. When the nitrogen injection volume is 1000 m^3^/h, the width of the spontaneous combustion zone in goaf is reduced by about 51 m compared with that without nitrogen injection measures.

**Table 2:** Distribution of spontaneous combustion zone in goaf with different nitrogen injection volume.

nitrogen injection volume/(m^3^∙h^−1^)	*v*_max_ = 0.24 m/min	*C*_min_ = 7%	width/m
0	55 m	128 m	73
500	22 m	71 m	49
600	20 m	60 m	40
700	20 m	51 m	31
800	16 m	43 m	27
900	15 m	39 m	24
1000	13 m	35 m	22

**Figure 8:**
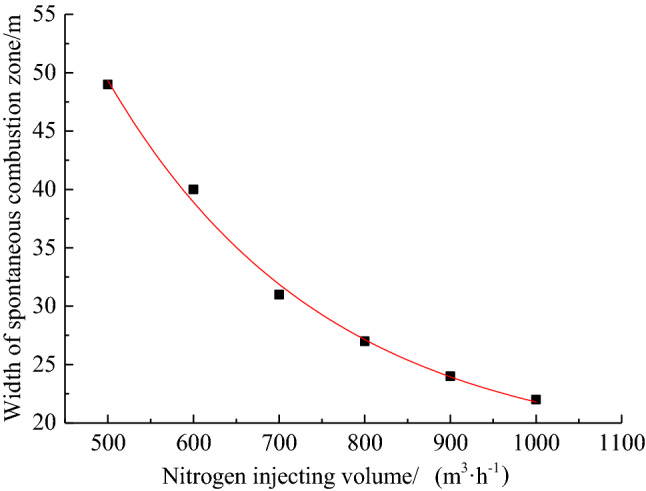
Fitting curve between nitrogen injection volume and width of spontaneous combustion zone.

According to the data in Table 2, Origin software is used to fit the data. The fitted curve of the width of the spontaneous combustion zone in the goaf with the variation of nitrogen injection volume was obtained, as is shown in Fig. 8. As known from Fig. 8, the width of the spontaneous combustion zone showed a negative exponential decreasing trend with the increase of the nitrogen injection volume. The following formula is the obtained fitting formula:

2 $$L_{1} = 230.20 \cdot \exp \left( { - Q_{N} /253.34} \right) + 17.35$$

3 $$v_{1} \tau \ge L_{1}$$

where *v*_1_ is the actual advancing speed of working face, m/d; $\tau$ is the shortest coal spontaneous combustion period, d; *L*_1_ is the width of spontaneous combustion zone, m; *Q*_N_ is the nitrogen injection volume, m^3^/h. The optimal nitrogen injection volume is 690 m^3^/h by calculation. The fitting equation can predict the required nitrogen injection volume at different propulsion speeds and the width of the spontaneous combustion zone in the goaf at different nitrogen injection volume.

## Temperature field distribution in goaf after nitrogen injection

### Effect of nitrogen injection location on temperature field in goaf

**Figure 9:**
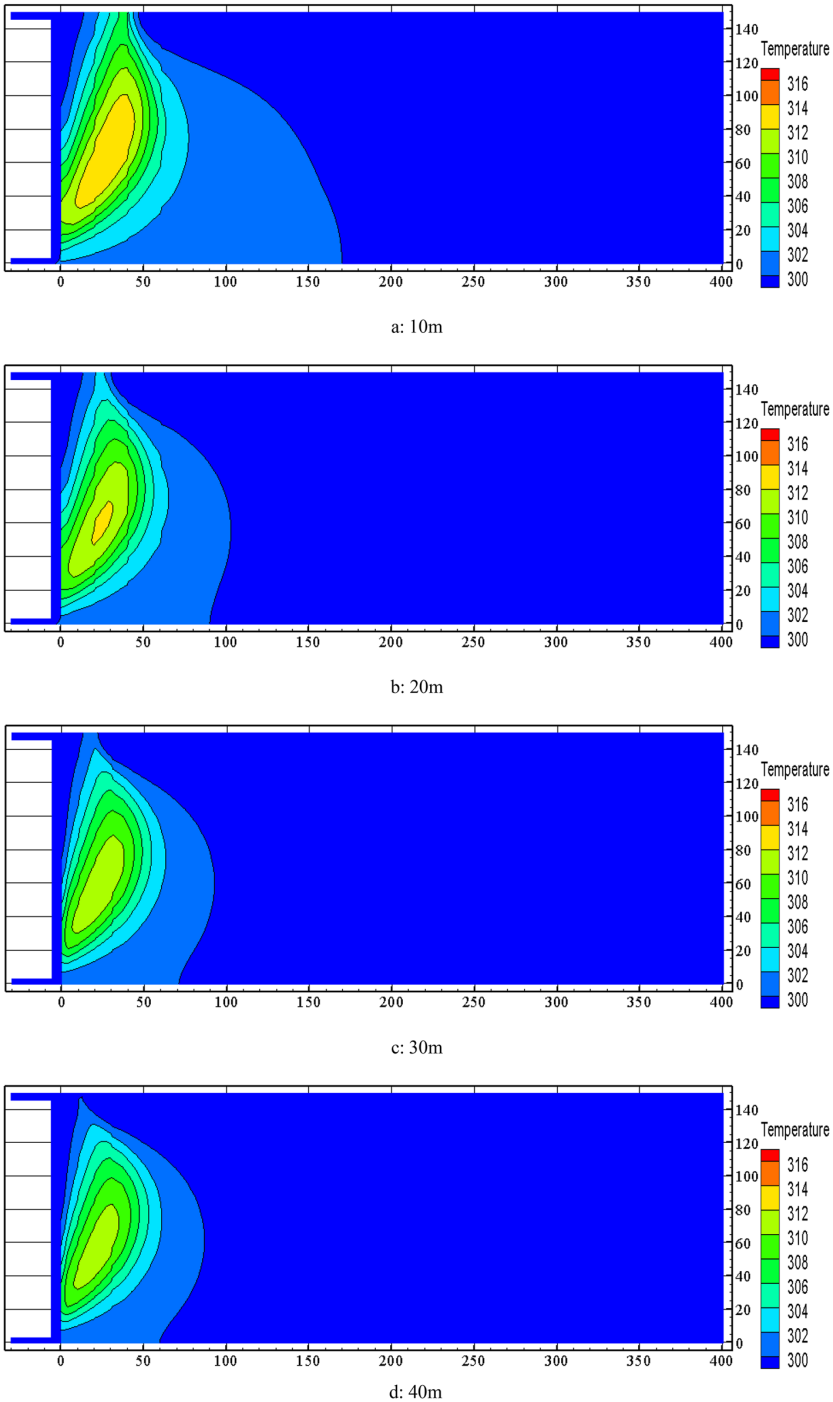

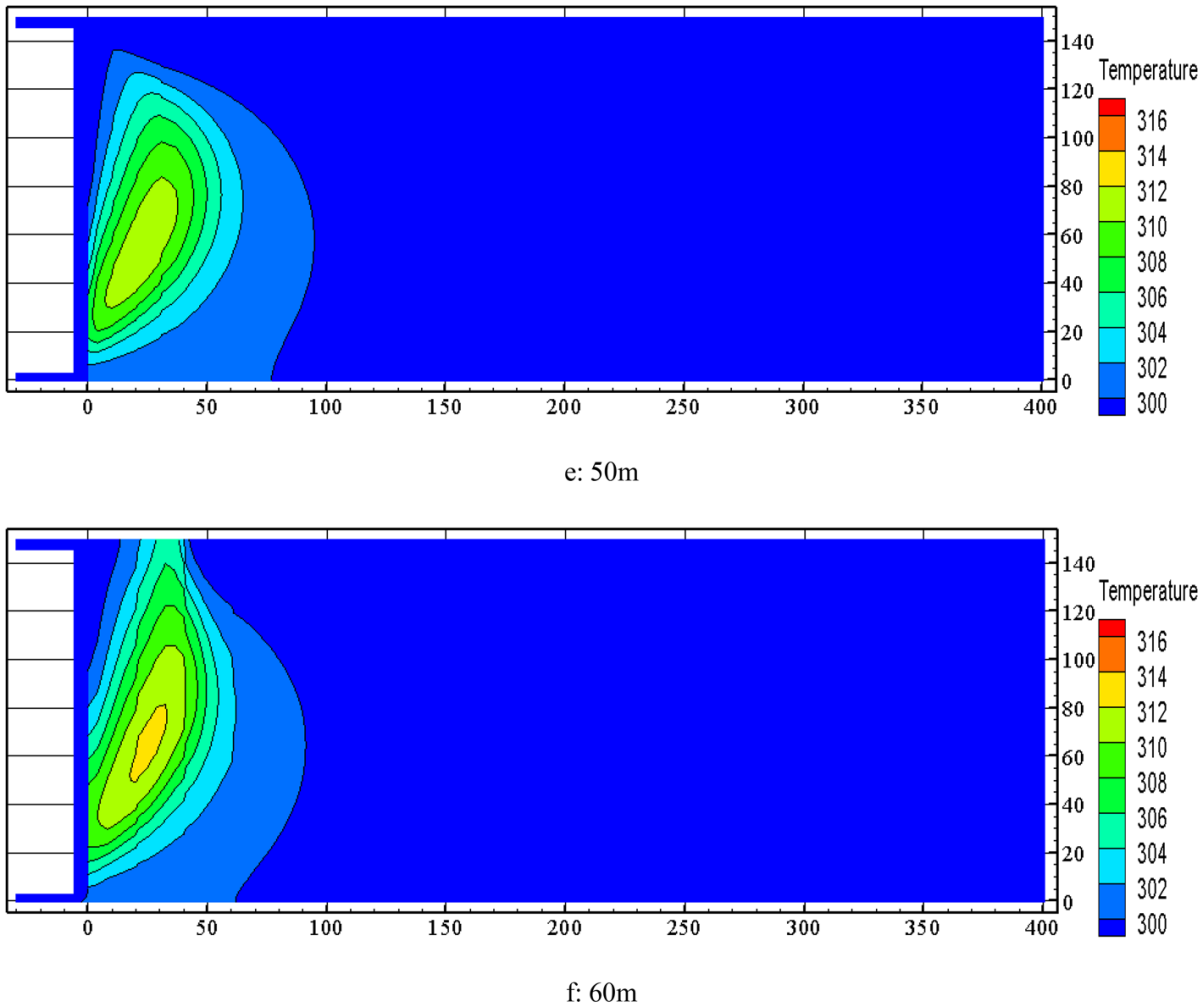
Effect of nitrogen injection location on temperature field in goaf.

FLUENT software was used to simulate the distribution of the temperature field in the goaf at the nitrogen injection locations of 10 m, 20 m, 30 m, 40 m, 50 m and 60 m when the nitrogen injection volume was 700 m^3^/h, and the results are shown in Fig. 9.

It is known from Fig. 9 that the high temperature zone in the goaf is obviously reduced after nitrogen injection, and temperature extreme value are significantly decreased. The temperature is reduced from 342.05 K to 309.95 K at nitrogen injection location of 40 m, which has the best effect on temperature rise suppression and greatly reduces the risk of spontaneous combustion in the goaf. The high temperature zone migrates to the middle of the goaf and the return air side. The oxygen in the inlet air side is significantly diluted by the nitrogen injection drive so that the heat production from coal oxygen in the area is reduced. The influence of the central and return wind side is relatively small. While the oxygen concentration of the central and return air side is higher than that of the inlet air side, and the heat production from the coal oxygen is relatively large. The temperature is higher than that of the inlet air side.

### Effect of nitrogen injection volume on temperature field in goaf

**Figure 10:**
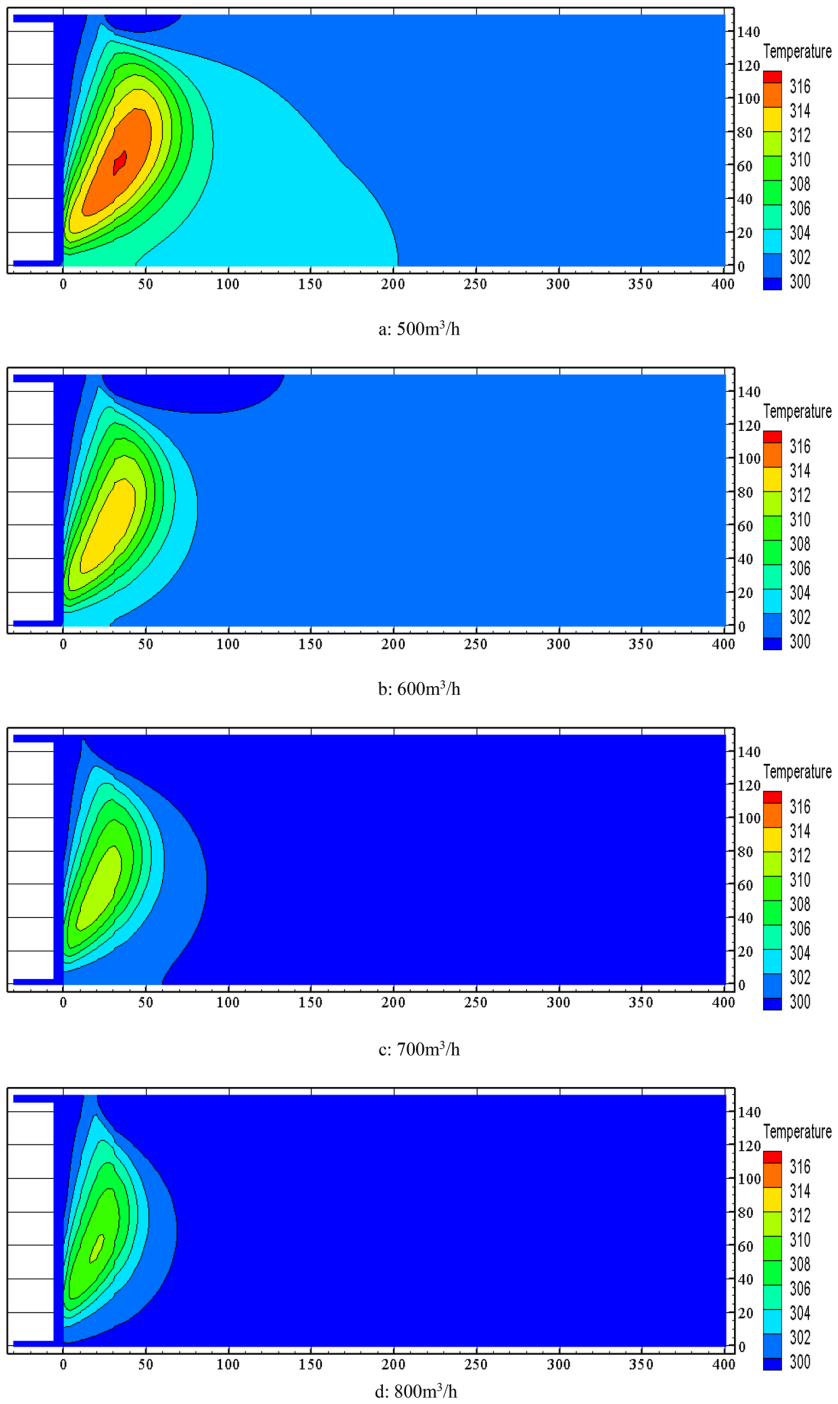

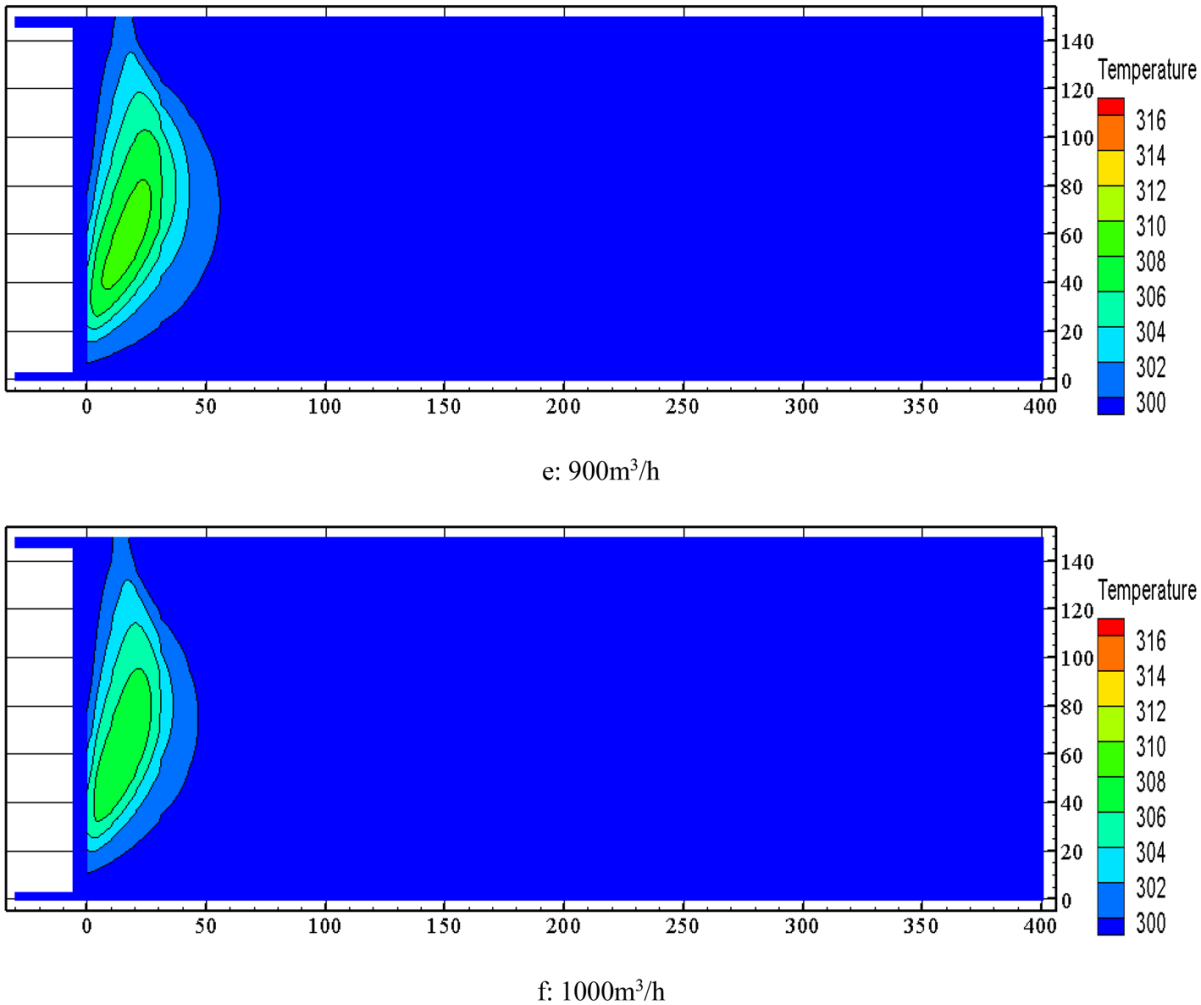
Effect of nitrogen injection volume on temperature field in goaf.

FLUENT software was used to simulate the distribution of temperature field in the goaf at nitrogen injection location of 40 m and nitrogen injection volume of 500 m^3^/h ~ 1000 m^3^/h. The simulation results of temperature contour distribution of 0.5 m section from the bottom plate are shown in Fig. 10. It is known from Fig. 10 that increasing the nitrogen injection volume in the goaf has an obvious inerting cooling effect. Both the area of high temperature zone and temperature extreme value are decreasing. The temperature extreme value decreases from 316.75 K to 306.35 K when the nitrogen injection volume increases from 500 m3/h to 1000 m3/h.

Comprehensive analysis of the above study shows that due to the large thickness of residual coal and more serious air leakage in the goaf of 10,101 fully mechanized top coal caving face in Baozigou coal mine, the temperature extreme value in goaf approaching 343.15 K during advancement of the working face. Nitrogen injection in the inlet air side of the goaf can play a good inerting and cooling effect on the goaf. When the nitrogen injection parameter is set to (40 m–1000 m^3^/h), the temperature extreme value of the goaf decreases by 308.85 K compared with that without nitrogen injection, which greatly reduces the risk of residual coal spontaneous combustion in the goaf.

## Fire-fighting technology of nitrogen injection and shot-off loss wind in goaf

### Nitrogen injection fire-fighting technology in goaf

FLUENT software was used to simulate and research the effects of nitrogen injection location and nitrogen injection volume on oxygen concentration, air leakage velocity and temperature in the goaf to optimize the nitrogen injection and fire prevention parameters. When the nitrogen injection location is 40 m and the nitrogen injection volume is 690 m^3^/h, it can meet the requirements of inerting and cooling in the goaf of 10,101 fully mechanized top coal caving face. When applying it, the nitrogen injection pipeline is buried in the goaf on the air inlet side of fully mechanized top coal caving face to inject nitrogen with reference to the parameter. The nitrogen production equipment is DT-700 type underground mobile molecular sieve nitrogen production device with nitrogen flow rate of 700 m^3^/h and nitrogen purity not less than 97%. The mobile nitrogen unit is arranged in the track roadway of a mining area. A 6-inch seamless steel pipe is selected as the nitrogen pipeline to transport nitrogen, with the steel pipes being connected to each other by flanges. The nitrogen transport pipeline is set up close to the outer coal wall of the air inlet roadway. The nitrogen injection pipe is buried at the back of the lower corner of the working face and the nitrogen is injected by buried pipe. When the nitrogen injection pipe enters the goaf 40 m, the valve can be opened for nitrogen injection, while the next nitrogen injection pipe is laid. When the first trip of the pipeline into the goaf 80 m can no longer inject nitrogen, and the second trip of nitrogen injection pipeline valve is opened for nitrogen injection.

### Fire-fighting technology of shot-off loss wind in goaf

**Figure 11:**
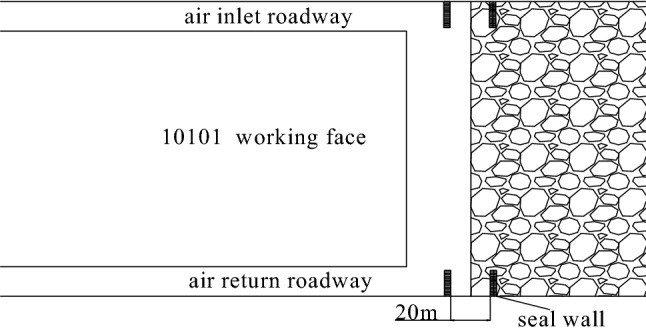
10,101 fully mechanized top coal caving face goaf seal wall layout location.

The large width of the spontaneous combustion zone in the goaf indicates that there are more serious air leakage. The seal wall is built at the corner of the inlet and return air roadways of the 10,101 working face to reduce the amount of air leakage in the goaf and the risk of spontaneous combustion. When nitrogen injection in the goaf is combined with construction of seal walls under the circumstances of changing the air leakage flow field, the effectiveness of nitrogen injection can be significantly improved. The air proof seal wall is arranged at the corner of inlet and return air roadways in the 10,101 working face, as is shown in Fig. 11. The seal wall is constructed by solid foam injection. The masonry height is the same as the mining height. The actual thickness of each seal wall is not less than 1.5 m to ensure tight sealing and no air leakage. The two seal walls are separated by 20 m. The seal wall built in the inlet and return air roadways is combined with the injection of gel in the goaf to achieve good effect on plugging air leakage in the goaf and controlling the air leakage from working face to goaf. Combined with the actual situation of the working face and the inlet and return air roadways, it is more difficult to build a seal wall over 8 m, so the length of the seal wall is set at 8 m.

### Effect analysis of spontaneous combustion prevention

**Figure 12:**
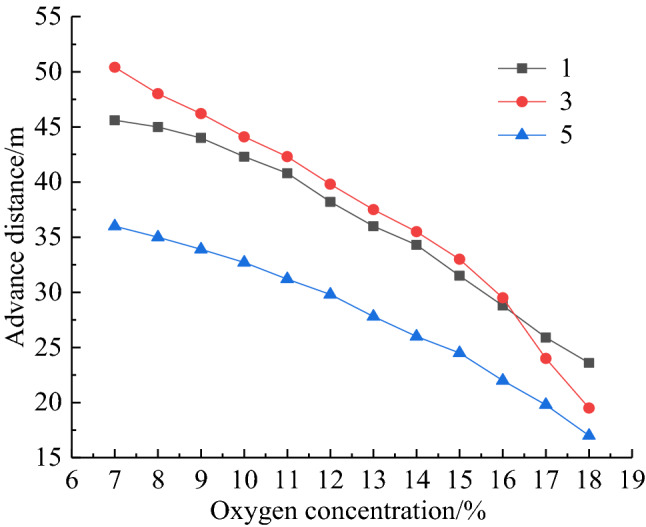
Change curve of oxygen concentration in goaf after nitrogen injection.

To investigate the accuracy of the numerical simulation of nitrogen injection in the goaf and the application effect of nitrogen injection for fire prevention, the distribution of spontaneous combustion "three zones" in the goaf was monitored during the period when only nitrogen injection for fire prevention was used in the goaf of 10,101 fully mechanized top coal caving face. It is shown from Fig. 12 that the width of the spontaneous combustion zone at measuring point 1 is 22 m, that at measuring point 3 is 30.9 m, and that at measuring point 5 is 19 m. The measured results are basically consistent with the maximum width 31 m of the spontaneous combustion zone from the simulation, which verifies the reliability of the simulation results of nitrogen injection in the goaf.

**Figure 13:**
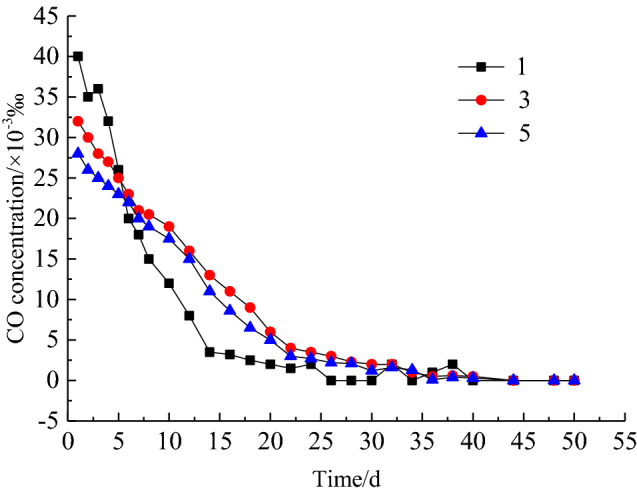
Change of CO concentration at each measuring point.

**Figure 14:**
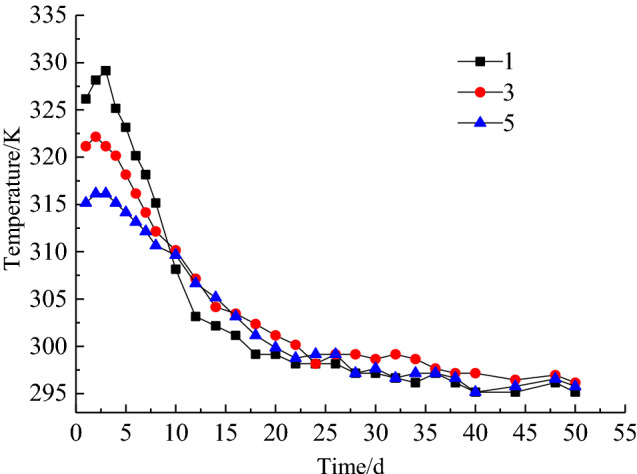
Temperature change curve of each measuring point.

To grasp the application effect of comprehensive fire prevention and suppression technology in the goaf of 10,101 working face, the measured data of CO concentration and temperature at every measuring point during the engineering application of comprehensive fire control measures are shown in Fig. 13 and Fig. 14 respectively. From Fig. 13 and Fig. 14, at the beginning of application of comprehensive fire control measures, the CO concentration at the location of measuring point 1, 3 and 5 are 40 × 10^−3^‰, 32 × 10^−3^‰ and 28 × 10^−3^‰ respectively. After 5 days, there is significant reduction trend in the CO concentration and the effect of spontaneous combustion prevention in the goaf begins to appear. After 30 days, CO concentration of 0 × 10^–3^‰, 2 × 10^−3^‰ and 1.2 × 10^−3^‰ are detected at measuring point 1, 3 and 5 respectively. At the same time, a significant decrease in temperature also occurs at each measuring point, with temperature at measuring point 1, 3 and 5 decreasing from 329.15 K, 322.15 K and 316.15 K to 295.15 K, 296.15 K and 295.65 K. Temperature stabilization time at measuring point 1 is significantly earlier than that at measuring points 3 and 5. After that, the CO concentration remains at 0 during recovery time, and no further significant high temperature points are monitored in the goaf. The engineering application results show that using comprehensive measures has achieved successful control of residual coal spontaneous combustion in the goaf of 10,101 working face.

## Conclusions

1. A three-dimensional dynamic nitrogen injection multi-field coupled numerical model of the goaf was established. The effect of the nitrogen injection position, nitrogen injection flow and other parameters on the oxygen concentration of the goaf and the velocity of air leakage was stimulated through FLUENT software to obtain the distribution range of the "three zones" of spontaneous combustion in the stable stage and optimize the nitrogen injection fire prevention and control parameters. The inerting requirements of the goaf could be met when the nitrogen injection depth was 40 m and the nitrogen injection volume was 690 m^3^/h. The width of the spontaneous combustion zone was 31 m.
2. After nitrogen injection in the goaf, the high temperature zone and the temperature extreme value was significantly reduced. The best suppression of temperature rise in the goaf was achieved at a nitrogen injection depth of 40 m, reducing from 342.05 K without nitrogen injection to 309.95 K. The temperature extreme value decreased from 316.75 to 306.35 K when nitrogen injection volume increased from 500 to 1000 m^3^/h. Compared with that without nitrogen injection, the temperature extreme value of goaf was reduced by 308.85 K when the nitrogen injection parameter was set to (40 m–1000 m^3^/h).
3. The comprehensive prevention and control measures of spontaneous combustion in goaf were applied in the goaf of 10,101 fully mechanized work face. After 30 days, the CO concentration of measuring point 1, 3 and 5 decreased from 40 × 10^–3^‰, 32 × 10^–3^‰, 28 × 10^–3^‰ to 0 × 10^–3^‰, 2 × 10^–3^‰ and 1.2 × 10^–3^‰, the temperature of the corresponding measuring point decreased from 329.15 K, 322.15 K, 316.15 K to 295.15 K, 296.15 K and 295.65 K. The spontaneous combustion of residual coal in the goaf has been successfully managed.

## Supplementary Information

Supplementary Information.

## Data Availability

All data generated or analysed during this study are included in this published article and its supplementary information files.
