# Supplementary material for: Study on multi field coupling numerical simulation of nitrogen injection in goaf and fire-fighting technology
Source: Sci Rep. 2022 Oct 17;12:17399. doi: 10.1038/s41598-022-22296-9 (PMC9576797; doi:10.1038/s41598-022-22296-9)
Supplement: Supplementary file 1 — Supplementary Information. [file 41598_2022_22296_MOESM1_ESM.docx]

I declare that all data generated or analysed during this study are included in this published article and its supplementary information files.

The size of geometric model is determined according to actual situation of 10101 fully mechanized top coal caving face of Baozigou coal mine. The length of working face is 150m, and the height is total height of caving zone and fracture zone of 50m. The length of goaf is taken to be 400m as an illustration. In order to reflect the actual situation of the ventilation resistance of the working face and simplify the model, the section of working face is 6m×3.2m and the section of return air roadway is 3.6m×3.2m and the section of air inlet roadway is 4.5m×3.2m, with the length of 25m. The three-dimensional geometric model is shown in Fig. 3. The solution is based on a unit time step of 1d, with an average daily advance speed of 3.6m/d as the increasing rate of goaf length. With the advancement of working face, the goaf is gradually extended. The grid is automatically updated by the CFD software's dynamic grid model, thus reflecting the continuous advance of working face. Space moving coordinate system forms a new grid at a 3.6m/d advance rate. According to the characteristics of dynamic grid model, the boundary conditions move as the working face advances dynamically. Temperature of the newly emerged coal rock is set as the initial temperature, after which the temperature continues to rise with the compound action of coal-oxygen.

When selecting the main calculation conditions and parameters, it is necessary to follow the actual situation on site. The air inlet roadway of working face is set as inlet boundary. The air return roadway is set as free boundary. The nitrogen injection port is set as the velocity inlet and the concentration of nitrogen is taken to be 97%. Actual measured air flow temperature in air inlet roadway is 18.6°C. The oxygen concentration is 20.9% and the actual wind speed at the working face is 1.62 m/s. The average air density is 1.225kg/m^3^. The air viscosity coefficient is taken to be 1.7894×10^-5^kg/(ms). The diffusion coefficient of gas is 2.88×10^-5^m^3^/s and the loosening coefficient is set to 1.5.

Range of spontaneous combustion "three zones" in goaf of different measuring point

| measuring point | cooled zone/m | spontaneous combustion zone/m  至工作面距离/m | suffocating zone/m  至工作面距离/m |
| --- | --- | --- | --- |
| 1 | 0~62 | 62~132 | >132 |
| 2 | 0~52 | 52~112 | >112 |
| 3 | 0~40 | 40~95 | >95 |
| 4 | 0~38 | 38~93 | >93 |
| 5 | 0~38 | 38~90 | >90 |

Distribution of spontaneous combustion zone in goaf with different nitrogen injection volume

| nitrogen injection volume/(m^3^∙h^-1^) | *v*_max_=0.24m/min | *C*_min_=7% | width/m |
| --- | --- | --- | --- |
| 500 | 22m | 71m | 49 |
| 600 | 20m | 60m | 40 |
| 700 | 20m | 51m | 31 |
| 800 | 16m | 43m | 27 |
| 900 | 15m | 39m | 24 |
| 1000 | 13m | 35m | 22 |
